# Daily and Seasonal Activity Patterns of the Endemic Balkan Snow Vole (*Dinaromys bogdanovi*) Within a Sympatric Small Mammal Community

**DOI:** 10.3390/ani16203236

**Published:** 2026-10-15

**Authors:** Duje Lisičić, Marko Glogoški, Ivana Selanec, Ivan Budinski, Toni Safner, Maša Ljuština, Tomislav Gojak, Vesna Benković

**Affiliations:** 1 Department of Animal Physiology, Faculty of Science, University of Zagreb, Rooseveltov trg 6, 10000 Zagreb, Croatia; duje.lisicic@biol.pmf.unizg.hr (D.L.); vesna.benkovic@biol.pmf.unizg.hr (V.B.); 2 Association BIOM, Matića 12, 21230 Sinj, Croatia; ivana.selanec@biom.hr (I.S.); ivan.budinski@biom.hr (I.B.); 3 Department of Plant Breeding, Genetics and Biometrics, Faculty of Agriculture, University of Zagreb, Svetosimunska 25, 10000 Zagreb, Croatia; tsafner@agr.hr; 4 Institute for Environment and Nature, Ministry of Environmental Protection and Green Transition, Radnička cesta 80, 10000 Zagreb, Croatia; mljustenator@gmail.com; 5 Association Hyla, Lipovac I 7, 10000 Zagreb, Croatia; tomislav.gojak@hhdhyla.hr

**Keywords:** activity patterns, biodiversity hotspot, Balkan Peninsula, camera trapping, endemic species, Mediterranean karst, small mammals, temporal niche partitioning

## Abstract

Understanding the temporal ecology of endemic species is important for conservation planning in biodiversity hotspots during global climate change and habitat alteration. We used three years of camera-trap monitoring across two deployment periods to quantify daily and seasonal activity patterns of the endemic Balkan snow vole (*Dinaromys bogdanovi*) and sympatric terrestrial small mammals in Biokovo Nature Park, Croatia, within the Western Balkan glacial refugia. *Dinaromys bogdanovi* was predominantly nocturnal, with minor but consistent diurnal activity, and shifted from multimodal nocturnal activity in spring, summer and autumn to a unimodal winter peak. Temporal niche differentiation occurred between *D. bogdanovi* and the sympatric small mammals during some seasons. These differences were associated with nocturnal peak timing and the greater diurnal activity of *D. bogdanovi*. In summer, the detected taxa showed greater overlap in predominantly nocturnal activity, consistent with a possible influence of thermal conditions on surface activity, although temperature was not measured. We also provide quantitative activity data for the geographically isolated Dinaric population of *Eliomys quercinus*. This study demonstrates the value of long-term camera trapping for monitoring cryptic small mammals and provides a baseline for evaluating future changes in activity, habitat use and community interactions, supporting conservation planning for endemic species facing climate change and anthropogenic pressures.

## Simple Summary

Species found only in small geographic areas contribute to regional biodiversity, but gaps in basic knowledge about their ecology can make conservation planning difficult. Over three years, we used motion-activated cameras to study the daily and seasonal activity of the Balkan snow vole, a small endemic mammal restricted to the western Balkans, and other small mammals living in the same habitat in Biokovo Nature Park, Croatia. The Balkan snow vole remained active throughout the year, predominantly at night, with low levels of daytime activity in spring, autumn, and winter. Its activity patterns varied among seasons and differed from those of other small mammals in spring, autumn, and winter. In summer, activity was concentrated at night, and we detected no significant differences between the vole and the other groups studied. The study also provides new information on the activity of the poorly known Dinaric population of the garden dormouse. Our findings demonstrate the value of long-term camera monitoring for studying small mammals that are otherwise difficult to observe. This information can help guide wildlife surveys, provide a baseline for detecting future changes in activity, and inform conservation planning for regional biodiversity.

## 1. Introduction

Endemic species constitute important components of local ecosystems, exhibiting specialized adaptations to environmental conditions and maintaining unique ecological relationships within their native habitats [1,2]. Endemics often serve as flagship taxa for regional biodiversity, representing unique evolutionary lineages restricted to specific geographic areas [3]. However, their restricted geographic ranges and specialized requirements make endemic species vulnerable to anthropogenic disturbances [4]. Consequently, endemics face higher extinction risk compared to non-endemic congeners, particularly under current scenarios of habitat loss and climate change [5,6]. Yet, for many endemic species, data on fundamental biological parameters are limited, hindering evidence-based conservation planning [7,8]. Understanding basic ecological parameters, such as niche occupancy, is therefore essential for informing targeted conservation strategies [9,10].

The Balkan snow vole (*Dinaromys bogdanovi*) is an endemic herbivorous rodent of the western Balkans, the only living representative of the genus and of an ancient arvicoline lineage that has evolved independently of the remaining living voles and lemmings since the late Miocene [11,12,13]. It is recognized as a vulnerable endemic species according to Rabinowitz’s ‘seven forms of rarity’ model [12] and therefore has been classified as vulnerable (VU) according to the IUCN Red List [14], is listed as an Annex II species in the Habitats Directive [15] and is strictly protected in Croatia [16]. *Dinaromys* is a habitat specialist, restricted to rocky karst terrain characterized by deep limestone fissures, crevices, and subterranean systems. This habitat specialization restricts its distribution to karst areas of the Dinaric Mountain range, where it occurs from sea level to approximately 2200 m a.s.l., with highest densities reported at mid-to-high elevations [12,17,18,19]. The ecological role of *Dinaromys* within the Dinaric mountain range, a recognized European glacial refugium and biodiversity hotspot [20], remains poorly understood. As an herbivore, it may influence vegetation dynamics. It may also serve as prey for mesopredators and potentially function as a reservoir for zoonotic pathogens, but all this remains strictly speculative. Research on *Dinaromys* ecology remains limited, with existing studies focusing predominantly on phylogenetics and phylogeography [11]. To our knowledge, only one field ecological study in nature has estimated population density in *Dinaromys*, based on June and September observations at Mt. Bjelašnica in Central Bosnia [21]. That study reported that 85% of traps were activated at night, suggesting predominantly nocturnal activity. There are indications of year-round activity, but data on temporal activity patterns, their seasonal variations, and potential temporal niche partitioning with sympatric small mammals are essentially absent.

Temporal activity patterns dictate when an animal can optimally access resources while avoiding predators and competitors, thereby defining its unique temporal niche [22,23,24]. These behavioural rhythms are under the influence of endogenous regulation but can fluctuate substantially across both daily and seasonal scales, driven by changing environmental conditions, resource availability, and interspecific interactions [23,24,25,26]. Characterizing these dynamic patterns is therefore crucial; it provides the essential baseline data required to design accurate survey protocols, investigate the mechanisms of species coexistence, and evaluate a species’ vulnerability to anthropogenic disturbances [27,28].

To address these knowledge gaps, we used camera trapping to quantify the daily and seasonal activity patterns of *D. bogdanovi* and sympatric small mammals over three years in the central part of the species’ distribution range. Specifically, we aimed to (1) characterise daily and seasonal variation in the activity of *D. bogdanovi* and (2) examine its activity patterns in relation to those of sympatric terrestrial small mammals.

## 2. Materials and Methods

### 2.1. Study Area

**Figure 1:**
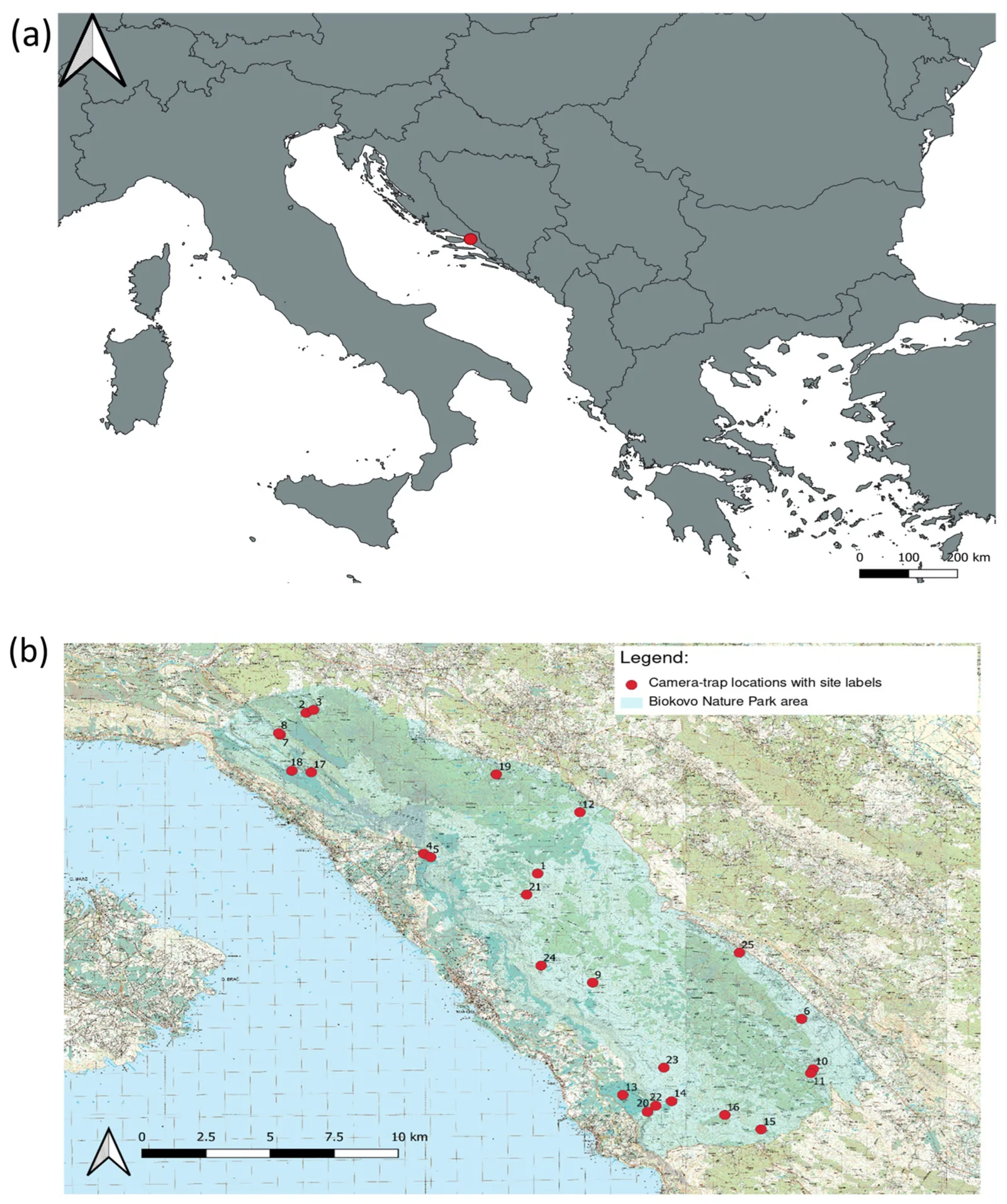
(**a**) Location of Mt. Biokovo in the Eastern Adriatic shoreline. (**b**) Camera-trap locations (red dots) for the Balkan snow vole (*Dinaromys bogdanovi*) within Biokovo Nature Park (green area): (1—Sveti Jure, 2 and 3—Bartulovići, 4 and 5—Bast, 6—Družjanići, 7 and 8—Gornja Brela, 9—Lađena, 10 and 11—Marasi, 12—Milići, 13 and14—Poganovac, 15 and 16—Prosika, 17 and 18—Sokolove Staje, 19—Stanići, 20—Topnički put, 21—Amfora, 22—Staza,23—Vilovik, 24—Vošac, 25—Župa). Adapted from [30].

The study was conducted in Biokovo Nature Park (longitude 17°3.74379′; latitude 43°19.75957′), Croatia, which encompasses Mt. Biokovo that stands in the central part of the Dinaric Mountains (Figure 1a). The park extends over an area of 193.271 km^2^ with its highest peak at 1762 m. The southern slopes of the mountain extend to sea level to form the Adriatic shoreline. Mt. Biokovo is a typical Dinaric Mountain, composed primarily of limestone and dolomite that form an abundance of typical karst structures, such as karrens, crevices, caves, and cliffs. The climate ranges from typical Mediterranean at lower elevations to more continental and montane towards the mountain ridges and peaks. Consequently, depending on the altitude and climatic conditions, vegetation zones vary from Mediterranean to alpine. Vegetation cover consists mostly of broad-leaved deciduous woodland (45% of total cover), scrub and garrigue (25%), and, to a lesser extent, Mediterranean or mountain grasslands (14%), while open rocks and scree comprise 9% of the total area [29].

In addition to *D. bogdanovi*, other endemic rock-dwelling vertebrates can be found in the area: sharp-snouted rock lizard (*Dalmatolacerta oxycephala*), Mosor rock lizard (*Dinarolacerta mosorensis*) or chamois (*Rupicapra rupicapra balcanica*). The small terrestrial mammal fauna is comprised of insectivores such as shrews (*Crocidura suaveolens*, *C. leucodon* and *Suncus etruscus*) and the northern white-breasted hedgehog (*Erinaceus roumanicus*), and an array of rodents such as wood, yellow necked and western broad-toothed field mouse (*Apodemus sylvaticus*, *A. flavicollis* and *A. epimelas*), garden dormouse (*Eliomys quercinus*), edible dormouse (*Glis glis*), and small carnivores like the least weasel (*Mustela nivalis*) and stone marten (*Martes foina*), while black and brown rat (*Rattus rattus* and *R. norvegicus*), and house mouse (*Mus musculus*) can be found near human settlements [17,31]. Information on the field biology for many of these species is scarce, both in the study area and throughout the Dinaric Mountains.

### 2.2. Data Collection

**Figure A1:**
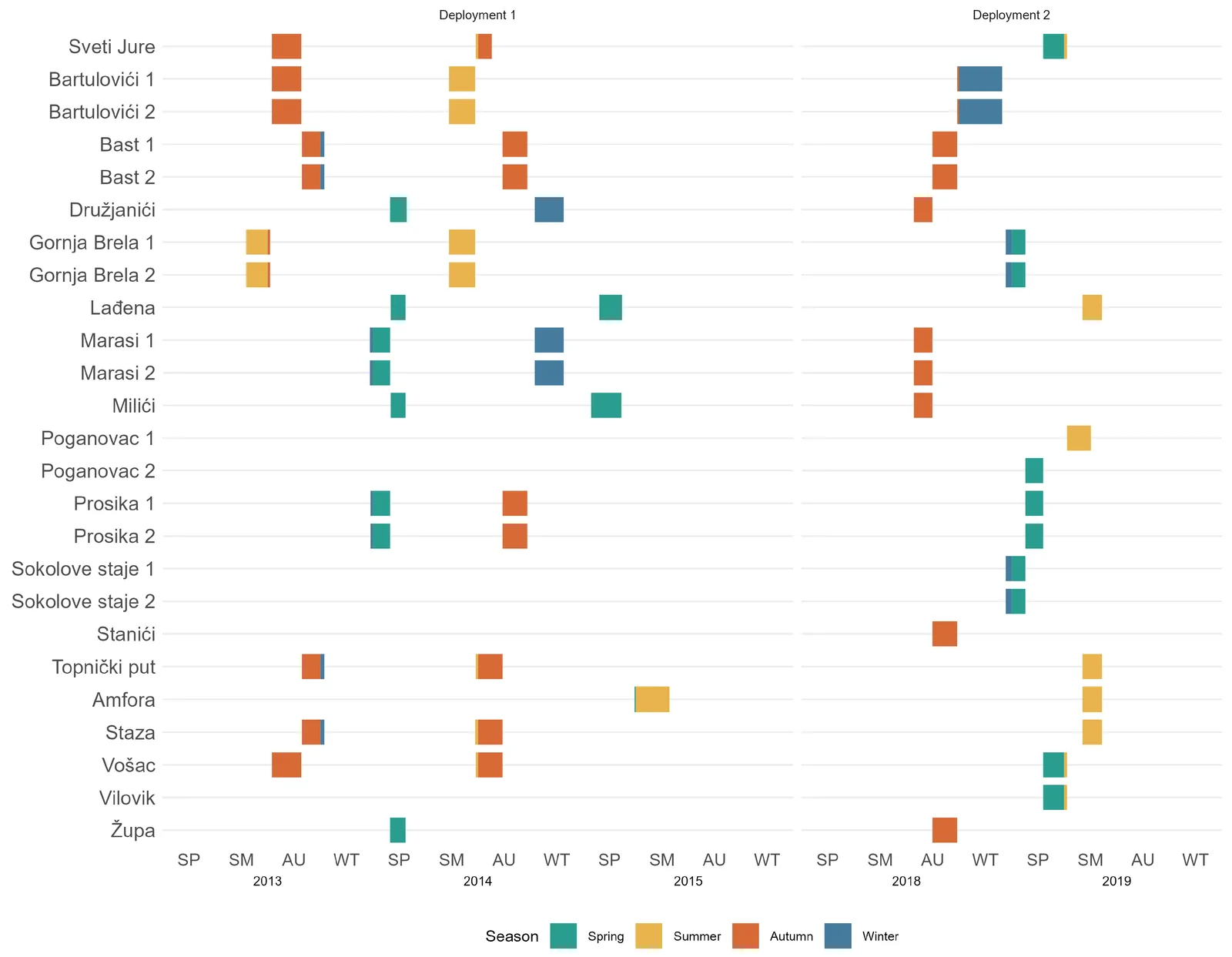
Camera-trap deployment schedule across the 25 sampling locations during the two deployment periods. Bars indicate the periods when a camera was operating at each location. SP—spring, SM—summer, AU—autumn, WT—winter.

The study was conducted from June 2013 to July 2015 and from September 2018 to August 2019, corresponding to three annual cycles of sampling across two deployment periods. Camera traps (Ltl Acorn 520a, Zhuhai Ltl Acorn Electronics Co., Ltd., Zhuhai, China) with an infrared system for night recording were deployed at 25 locations distributed throughout the park. Locations were distributed throughout the park area and were selected to cover major vegetation zones and elevations found within the park to ensure representative coverage across the park area (Figure 1b and Figure A1). As the focus of this study was *D. bogdanovi*, microlocations were selected to include the most suitable habitat for this karst-associated species, avoiding unsuitable sites such as dense forests or open, rock-free meadows. The focal distance of each camera trap was set to 30 cm to 1 m, photo resolution to 12.0 megapixels, and video resolution to 640 × 480 pixels per frame. Camera mode differed among locations, recording three still photographs, a 15 s video, or both per trigger. A paired-camera comparison has reported similar activity estimates from expert-classified photographic and video records [32]. Camera traps were positioned at ground level, between rocks or tree trunks, where the movement of small mammals was expected. After approximately one month of operation, individual camera traps were checked, the data downloaded, and the traps moved to another location for a new sampling cycle. Using this regime, total sampling effort across the selected locations was 2504 camera–days (Table A1).

After preliminary inspection of the obtained data, animals in the photographs were determined to the lowest taxonomical unit possible, and records of selected species or categories were retained for further analysis. In the final dataset, we retained observations of small rodents and insectivores that share the same terrestrial niche with *D. bogdanovi*. Unidentifiable or blurred records and species that were rarely recorded were excluded from further analysis. For analyses, the observations were grouped into four different categories: (1) *D. bogdanovi*, species clearly discriminates from others and the focus of this study, a herbivorous karst specialist; (2) *E. quercinus*, another species clearly discriminate from others, an omnivore-carnivore climber; (3) the *Apodemus* species group (*Apodemus sylvaticus*, *A. flavicollis,* and *A. epimelas*), omnivore–granivore species of similar morphological appearance, and (4) the shrew group (*Crocidura suaveolens*, *C. leucodon,* and *Suncus etruscus*), all small insectivores. Species were pooled into groups for two reasons: similar species (like *Apodemus* species or shrews) are difficult to identify to the species level based only on the captured photos or videos, and these groups of similar species all share certain morphological and ecological similarities [33,34]. Accordingly, the activity curves for *Apodemus* and Soricidae reflect pooled detections and should not be interpreted as species-specific activity patterns. Because camera locations were selected primarily on the basis of habitat suitability for *D. bogdanovi*, comparisons with sympatric taxa refer to individuals detected within *D. bogdanovi*-oriented sampling sites.

### 2.3. Studied Species

*D. bogdanovi* is a herbivore that feeds mainly on vegetation and herbs, reaching a body weight of 30 to 82 g [19]. *Eliomys quercinus* (45–120 g body weight) is another European endemic, with fragmented populations scattered through southern and central Europe [33]. The Dinaric population is geographically separated from other ones, and it was recognised as *E. quercinus dalmaticus*, showing its association with the karst habitat [35]. Today this population has the status of a subclade of the “Italian” clade of *E. quercinus* [33,36,37]. An opportunistic rodent of a similar size is *A. epimelas* (body weight 28–56 g) that feeds on nuts, fruits, and herbs. This species is found in the dry rock habitats from the Western Balkans to Asia Minor [34]. Both *A. sylvaticus* (14–30 g) and *A. flavicollis* (22–56 g) are smaller, opportunistic rodents widespread throughout Europe, feeding on vegetables and grains with the occasional snail or insect added to the diet [34]. All shrews found in the area typically live in complex habitats and rarely venture into open areas, feeding on insects and other invertebrates. The shrews are much smaller than other small terrestrial rodents studied here, with body weights ranging from 2.5 to 15 g [33].

### 2.4. Data Analysis

To limit repeated records of animals lingering near a camera, we retained one detection per taxon per camera site within a 15 min interval. This duration is comparable to event windows used in small-rodent camera-trapping studies [38], although it is an operational filtering rule rather than evidence that successive records represent different individuals. All independent detections for each species or taxonomic group were pooled across the 25 camera sites before fitting activity-density models. This pooling was necessary because, for several taxa and seasons, the number of independent detections per site was too low to support robust site-specific activity models. Consequently, the resulting activity curves represent population-level patterns across the study area, averaged over site-level variation in elevation, vegetation, and microclimate, rather than site-specific activity schedules. Seasons were divided as meteorological seasonal dates for studied years (Spring: 1 March–31 May; Summer: 1 June–31 August; Autumn: 1 September–30 November; Winter: 1 December–28 February (or 29 February in a leap year)).

To allow comparisons of activities across seasons and to minimise the confounding effect of seasonal changes in daylight hours, for each recorded observation we calculated its solar time as the time difference (offset) from sunrise to the time of observation [39].

Each capture event (photo and video) was treated as a single observation (data point), and the temporal distribution of observations for each species was used to represent their daily activity.

Because of the time gap between two deployments (2013–2015 and 2018–2019), we compared seasonal activity in *D. bogdanovi* between deployment periods before pooling the data. For each season in each deployment, we fitted the Kernel Density Estimations (KDE) based on the solar times of observations as a non-parametric way to estimate the probability density function (PDF). Confidence intervals were estimated by resampling the data with 1000 bootstrap resamples. For each season, we then compared the obtained PDFs between the two deployments using a Wald test.

To test the seasonal variability in activity patterns of *D. bogdanovi*, we compared PDFs representing the activity in each season using the method described above and then tested the differences between the obtained PDFs using a Wald test.

To assess differences in seasonal activity patterns between *D. bogdanovi* and other selected species, we first estimated the PDFs for each species in each season and then compared the obtained activity patterns using a Wald test for the statistical difference between two or more activity level estimates. The confidence interval for each coefficient was calculated using a bootstrap method with 10,000 samples generated from a representative sample of the population [40]. All described statistical analyses were performed using R 4.5.0 [41] with packages ‘activity’ (version 1.3.4) [42] and ‘overlap’ (version 0.3.9) [43].

## 3. Results

**Figure A2:**
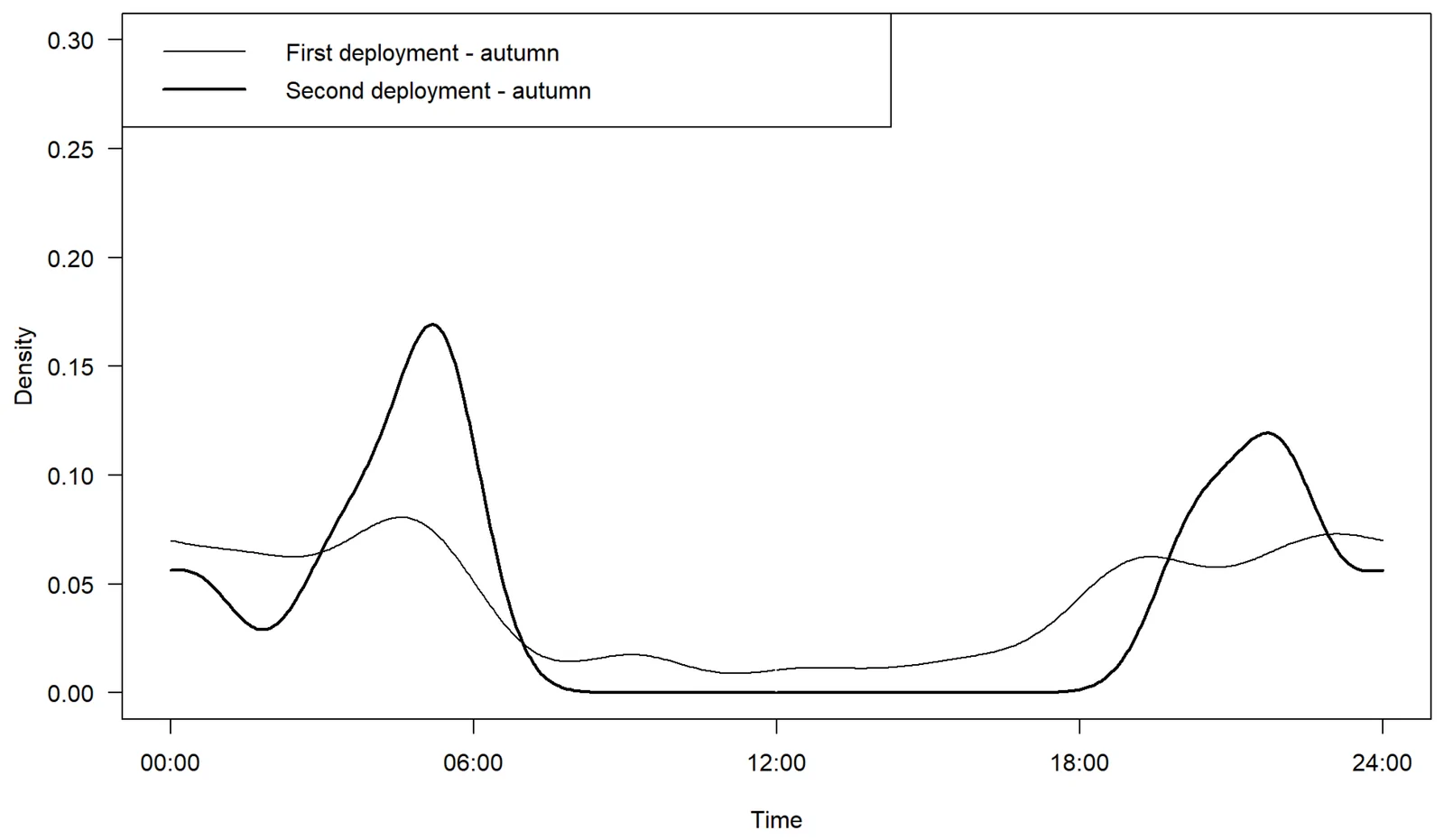
Kernel density estimates of autumnal daily activity distributions for *D. bogdanovi* in Biokovo Nature Park in two deployments (the first from June 2013 to July 2015 and the second from September 2018 to August 2019). X axis represents solar times (observation time with offset to the sunrise). The daily activity pattern was similar across both deployments.

No significant differences were detected in *D. bogdanovi* activity patterns between the two deployment periods during spring (difference = −0.12, *p* = 0.095), summer (difference = −0.10, *p* = 0.127), or winter (difference = 0.09, *p* = 0.297). In autumn, although overall activity differed significantly between deployments (difference = −0.27, *p* < 0.001), the general diel pattern remained consistent, with bimodal peaks around sunrise and after sunset and minimal activity during daylight hours (Figure A2). Consequently, these results indicate high temporal stability in activity rhythms, justifying the pooling of data across both deployments for subsequent analyses.

Over the course of the study, we obtained 1473 camera-trap detections across all target taxa. *Dinaromys bogdanovi* was detected at most sampled sites, with no detections at the only four (Družjanići, Sokolove staje 1 and 2, and Staza) of 25 sites (Figure 1b). The *Apodemus* group was the most frequently recorded category (46% of total detections), followed by *D. bogdanovi* (40%), Soricidae (11%), and *Eliomys quercinus* (4%). *Dinaromys bogdanovi*, the *Apodemus* group, and Soricidae were detected across all four seasons, whereas *E. quercinus* was completely absent during winter, which is consistent with the winter hibernation known for this species (Table 1).

**Figure 2:**
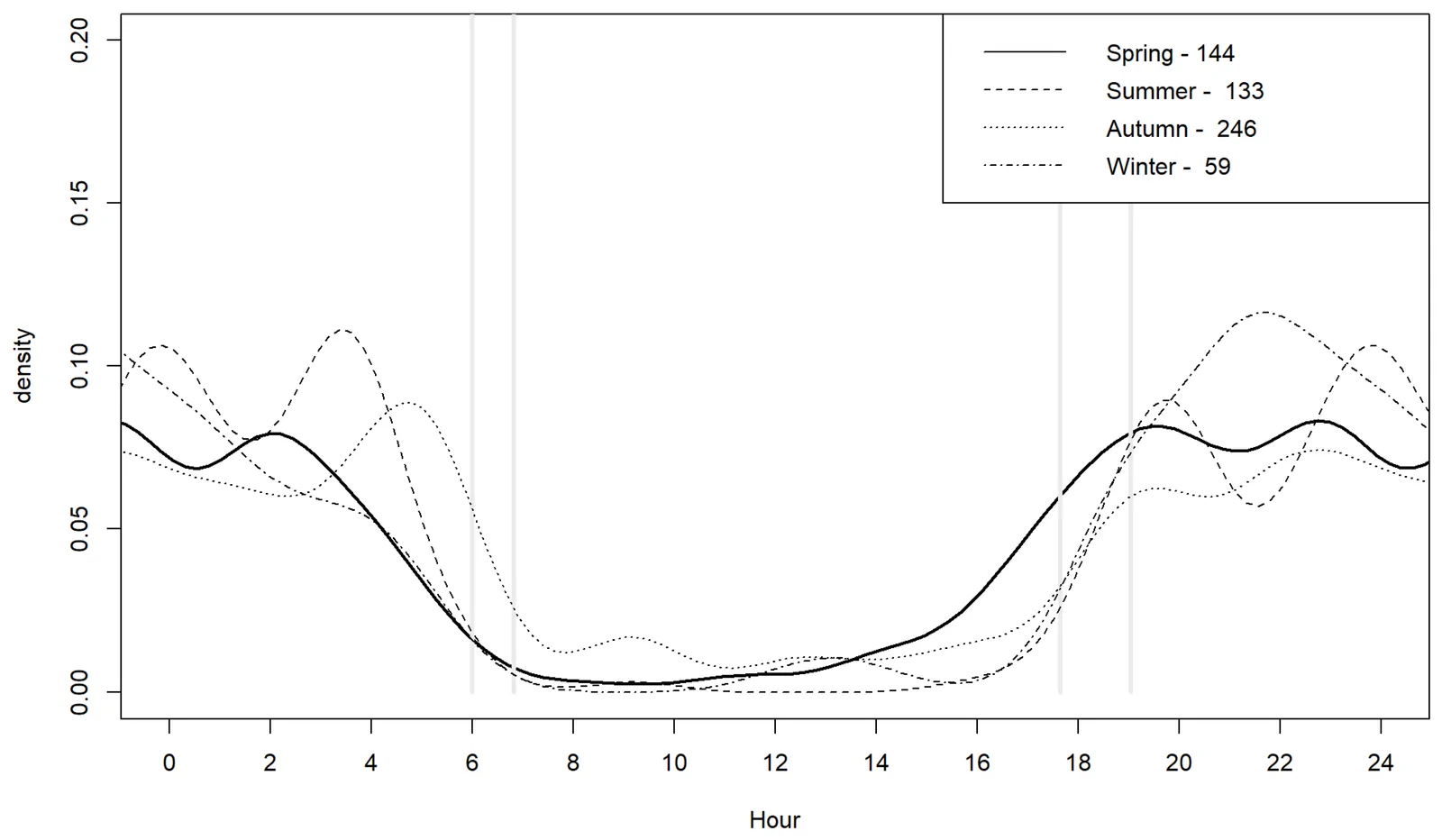
Kernel density estimates of daily activity distributions for *D. bogdanovi* in Biokovo Nature Park for each season. The *X*-axis represents solar times (observation time with offset to the sunrise). Grey vertical lines represent (in order): sunrise of the longest day, sunrise of the shortest day, sunset of the shortest, and sunset of the longest day of the year.

*Dinaromys bogdanovi* exhibited a pronounced preference for nocturnal activity while displaying minor activity during daylight hours (Figure 2). Activity typically declined 1–2 h before sunrise, reaching its baseline diurnal level at dawn. In the late afternoon (2–3 h before sunset), activity increased steadily, attaining peak nocturnal levels approximately one hour after sunset. Nocturnal activity was characterized by two to three distinct peaks during spring, summer, and autumn, whereas winter activity was unimodal; overall nocturnal intensity remained stable across seasons, with peak kernel density estimates ranging from 0.06 to 0.11. In contrast, daytime activity was consistently low, peaking at 0.02 in autumn and dropping to zero during summer. Pairwise comparisons using the Wald test indicated that seasonal activity patterns were largely consistent, with spring differing significantly only from summer and winter, likely reflecting the higher nocturnal peaks observed in the latter two seasons (Table 2).

**Figure 3:**
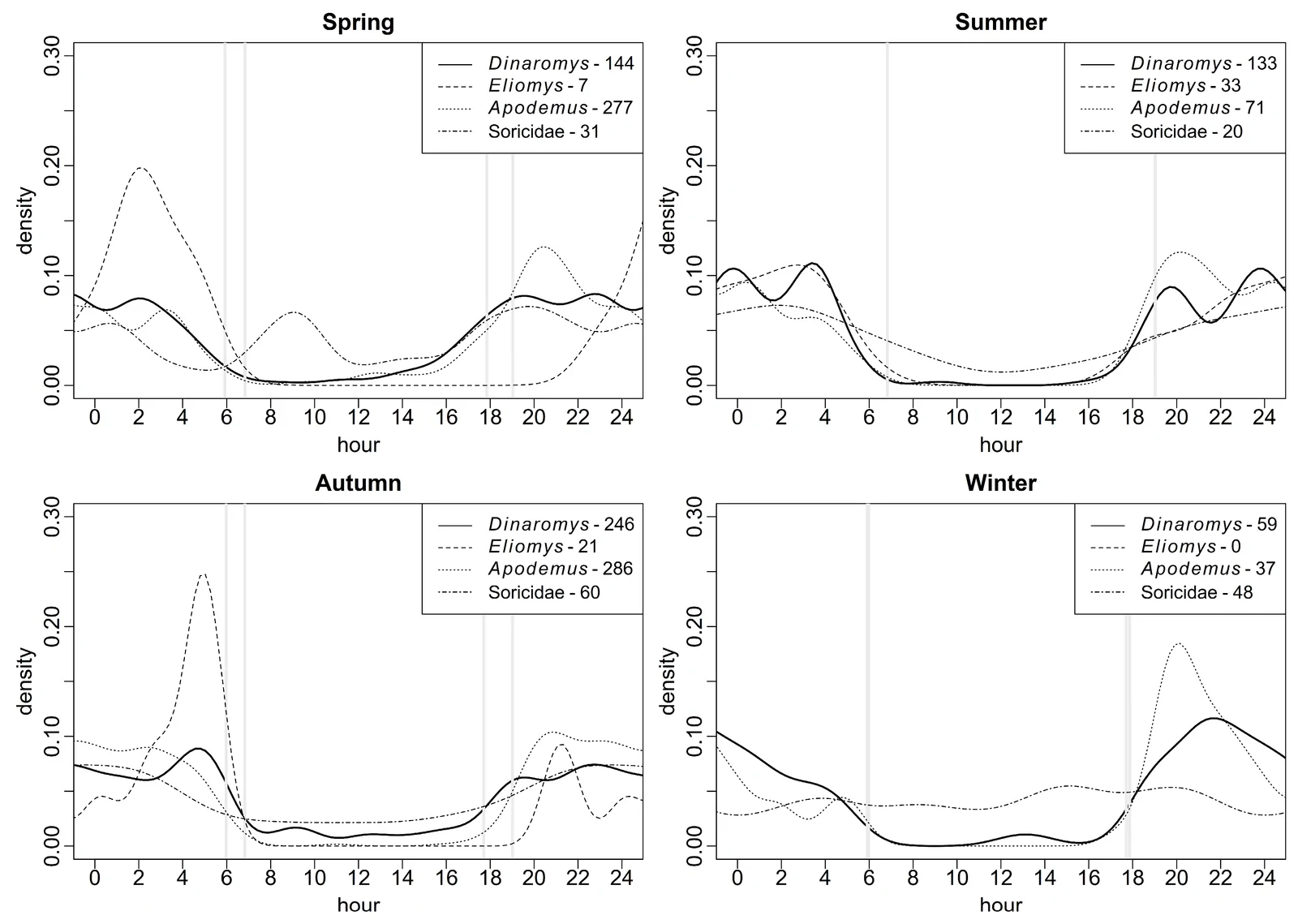
Kernel density estimates of daily activity distributions for four target species/groups in Biokovo Nature Park for each season. X axis represents solar times (observation time with offset to the sunrise). On each graph, grey vertical lines represent (in order): sunrise of the longest day, sunrise of the shortest day, sunset of the shortest, and sunset of the longest day of the season.

The estimated diel profiles of *D. bogdanovi* and the other sampled taxa showed differences in peak timing and the occurrence of daytime activity. Both *E. quercinus* and the *Apodemus* group exhibited strictly nocturnal activity, displaying virtually no diurnal movement (except *Apodemus* in the afternoon in spring), in contrast to *D. bogdanovi*, which maintained low levels of daytime activity (Figure 3). Soricidae, conversely, exhibited cathemeral activity across both day and night. Pairwise Wald tests revealed significant differences between *D. bogdanovi* and both *E. quercinus* and *Apodemus* in spring, between *D. bogdanovi* and *E. quercinus* in autumn, and between *D. bogdanovi* and both the *Apodemus* group and Soricidae in winter (Table 3). Specifically, the estimated activity distributions of *E. quercinus* showed sharper, higher nocturnal peaks than those of *D. bogdanovi* and no recorded late-afternoon activity during spring and autumn, although these patterns should be interpreted cautiously given the relatively small number of *E. quercinus* observations in these seasons. In spring, *Apodemus* group differed from *D. bogdanovi* by a prominent activity peak occurring two hours post-sunset. In winter, shrews displayed an almost uniform 24 h activity rhythm, whereas the *D. bogdanovi* and *Apodemus* group profiles were concentrated at night. No significant differences were detected between *D. bogdanovi* and any co-occurring taxon during summer, when activity profiles showed high interspecific concordance.

## 4. Discussion

Our results indicate that the endemic *D. bogdanovi* is predominantly nocturnal but exhibits low levels of diurnal activity. This pattern is consistent with evidence that rodents belonging to the Arvicoline subfamily often display flexible day–night activity, whereas murine rodents, including *Apodemus*, are predominantly nocturnal [44,45]. The diel activity pattern of *D. bogdanovi* also varied seasonally. Across spring, summer, and autumn, the species exhibited a multimodal nocturnal profile with two to three distinct activity peaks, whereas activity was concentrated into a single peak during winter. This seasonal compression may reduce exposure to harsh winter conditions and the associated thermoregulatory costs, because limiting surface activity during periods of sub-zero temperature can reduce metabolic expenditure in small mammals [24,25]. Low but consistent daytime and late-afternoon activity was recorded in spring, autumn, and winter, whereas no diurnal activity was recorded during summer. This pattern is consistent with seasonal flexibility in the timing of surface activity and with the tendency of small mammals to avoid the hottest parts of the day by using cooler microhabitats or shifting activity towards crepuscular and nocturnal periods [46,47,48,49]. However, because microclimatic and resource conditions were not measured concurrently, the mechanisms underlying these seasonal changes could not be determined.

At the broad diel scale, the activity of *D. bogdanovi* overlapped with that of other predominantly nocturnal members of the small-mammal community. Analysis of finer-scale activity distributions showed differences among taxa in spring, autumn, and winter. Unlike *D. bogdanovi*, the *Apodemus* group and *E. quercinus* displayed limited crepuscular but no diurnal activity (the exception is afternoon activity in *Apodemus* during spring), whereas shrews exhibited the cathemeral, polyphasic activity typical of Soricidae. More specifically, *E. quercinus* exhibited a sharp nocturnal peak in spring and two distinct peaks in autumn, whereas the *Apodemus* group peaked approximately two hours after sunset in spring. These differences illustrate that the studied rodents share a predominantly nocturnal activity period but differ in the timing of activity within the night. The apparent activity peaks of *E. quercinus* should be interpreted cautiously because spring and autumn samples were small. In summer, by contrast, activity was concentrated during the night with clear avoidance of daytime activity in all studied rodents. No significant differences were detected between *D. bogdanovi* and any co-occurring taxon during summer. The greater overlap in predominantly nocturnal activity during summer may be due to the constraints that high daytime temperatures impose on surface activity. Indeed, high daytime temperatures can limit diurnal activity, while shorter nights reduce the available nocturnal period [50,51,52], raising the possibility of reduced opportunities for temporal partitioning [49,53]. However, the observed summer activity pattern does not by itself demonstrate physiological thermal sensitivity or vulnerability to climate change. Seasonal differences in the pooled activity distributions need not arise solely from interactions among sympatric taxa. Daytime heat may make surface activity less favourable in summer, providing one possible explanation for the greater overlap in predominantly nocturnal detections, whereas shorter winter days and colder conditions may alter the timing of surface activity and contribute to the more concentrated winter peak of *D. bogdanovi*.

Beyond thermal conditions, activity timing in small-mammal communities can be shaped by local microclimate, microhabitat structure, vegetation, food availability, predation risk, and interspecific interactions [22,54,55,56,57]. For example, precipitation and snow cover may influence these patterns. In bank voles, nocturnal rainfall has been associated with a lower probability of activity at night [58]. A year-round camera-trap study recorded more small mammals at higher winter temperatures, although this relationship was significant only when snow cover was low [59]. These findings suggest that environmental conditions may contribute to seasonal changes in surface activity and detections, alongside changes in resource availability or interspecific interactions. Precipitation could affect exposure conditions and the availability of food or cover, while snow cover, where present, may change the accessibility and thermal conditions of rocky refuges and surface routes. Changes in photoperiod may also influence seasonal activity [60,61], although responses observed in other vole species cannot be assumed to apply directly to *D. bogdanovi*. These environmental influences may also differ among camera sites because of variation in elevation and vegetation. However, we did not measure local temperature, precipitation, snow cover, or resource conditions at our camera sites during monitoring, so we cannot determine their contributions to the observed patterns. The observed temporal differences should therefore be interpreted as seasonal patterns among taxa detected at the sampled sites, rather than as evidence that species interactions alone determine activity timing [27,62,63,64].

Continued global warming could further restrict the temporal windows available for surface activity and alter temporal overlap among sympatric species. Other studies suggest that progressive restriction of suitable niche may increase the potential for resource competition and favour ecologically flexible generalists over narrowly specialised endemic taxa [50,51,52]. Within this broader context, altered temporal overlap could increase the potential for competition between the habitat-specialist *D. bogdanovi* and widespread generalists such as *Apodemus* [65]. However, whether temporal overlap results in competition or competitive exclusion requires direct evidence and cannot be determined from activity data alone.

The study also addresses a separate knowledge gap by providing the first quantitative activity data for *E. quercinus* in the Dinaric Mountains. A European endemic, *E. quercinus* has undergone severe declines across much of its range [33,66]. Ecological information on the Dinaric population remains limited, with basic aspects of its autecology and temporal activity poorly documented [67].

Our records document the occurrence of *D. bogdanovi* across surveyed sites in Biokovo Nature Park in all four seasons, providing an empirical baseline for designing surveys and long-term monitoring. Its daily and seasonal activity patterns, examined alongside those of other small mammals sharing its habitat, offer a basis for evaluating future changes in activity timing and temporal overlap. The seasonal variation documented here shows why survey timing must be considered when interpreting detections and comparing records among seasons. These observations can support future assessments of whether climate warming, habitat alteration, or changes in community composition are accompanied by shifts in activity patterns. However, detection and activity data alone cannot establish causes of such shifts, demographic trends, food limitation, or competitive mechanisms. Furthermore, because live-trapping surveys of rare rock-dwelling rodents can be constrained by low capture probabilities and trap-associated stress [2,27], long-term camera trapping is particularly valuable in this context because it allows non-invasive, repeated sampling across seasons while reducing disturbance to elusive small mammals. When deployed systematically, camera traps can improve the detection of cryptic small mammals relative to conventional live trapping and provide information on species occurrence and distribution [68,69,70,71].

Yet, several limitations affect the interpretation of reported activity patterns. First, some seasonal sample sizes were small, particularly for *E. quercinus* in spring and autumn and for Soricidae in summer. Kernel density estimates for these groups may therefore be sensitive to individual observations, particularly in the timing and shape of apparent activity peaks. Second, because Apodemus and Soricidae each comprise multiple species, their activity distributions describe pooled groups whose species composition may vary seasonally. Differences in activity timing within these groups may therefore affect the group-level curves, which cannot be attributed to individual species. Third, because cameras were placed primarily in habitat suitable for *D. bogdanovi* and detections were pooled across 25 sites spanning a range of elevations and vegetation types, the resulting curves describe activity across the sampled habitat and may smooth or obscure site-specific differences in peak timing and activity breadth. Differences among taxa in the pooled activity distributions may partly reflect differences in habitat use rather than temporal differentiation alone. Consequently, detections of different taxa may have come from different subsets of cameras, each with its own local conditions. Because activity records were pooled across sites and habitat use was not analysed, we cannot determine whether the observed differences in activity timing also occur where taxa use the same sites. These patterns should therefore be interpreted as differences in activity among taxa detected in *D. bogdanovi*-oriented habitat, not as evidence of temporal partitioning independent of spatial habitat use. Because dietary overlap, microhabitat use and its conditions, demographic performance, and interspecific interactions were not measured directly, the present study cannot distinguish among them as explanations for the observed seasonal patterns.

Future work should prioritise year-round camera-trap monitoring paired with measurements of local microclimate and resource availability. Such monitoring would help determine whether thermal conditions influence activity timing and temporal overlap within this assemblage. Higher per-site sample sizes could also support tests of elevation- or habitat-specific activity patterns within the park. The exclusively nocturnal activity recorded in summer and the concentration of winter activity into a single peak provide a basis for investigating whether these seasons impose stronger constraints on surface activity. Such measurements could help identify potentially sensitive periods, although the activity patterns observed here do not establish seasonal vulnerability. Sampling across elevations and habitat types could also identify local conditions associated with continued occurrence, helping guide habitat protection and assess responses to habitat alteration. Combining these approaches with genetic sampling, dietary analyses, and occupancy or abundance models would help connect changes in activity and detection with population status, providing a stronger basis for conserving endemic mammals in Dinaric karst landscapes.

Beyond its local relevance, this study contributes to wider efforts to conserve endemic mammals by addressing persistent ecological knowledge gaps concerning geographically restricted and cryptic species. For such taxa, limited knowledge of distribution, population status, habitat requirements, and activity can constrain conservation assessment, monitoring design, and management [8,72]. Endemic taxa represent irreplaceable components of regional biodiversity, but their restricted ranges and specialised ecological requirements can increase vulnerability to environmental change and reduce their capacity to respond to changing conditions [9,73,74]. For endemic and relict species confined to patchy mountain systems, the consequences of narrow environmental tolerances may be compounded by increased contact with widespread generalists [5,6].

## 5. Conclusions

Long-term camera-trap monitoring revealed that *D. bogdanovi* is predominantly nocturnal but displays low levels of diurnal activity and marked seasonal variation in its activity distribution. Temporal activity patterns differed between *D. bogdanovi* and other sympatric terrestrial mammals in spring, autumn, and winter, whereas no significant differences were detected between *D. bogdanovi* and any co-occurring taxon during summer. Although the increased summer overlap is consistent with a possible influence of thermal conditions, our data do not identify its cause or demonstrate how activity patterns will respond to climate warming. Local thermal conditions, resource use, predation, and interspecific interactions would need to be measured to evaluate explanations for the observed seasonal patterns. The study also provides novel quantitative activity data for the poorly known Dinaric population of the European endemic *E. quercinus.* More broadly, this study illustrates how long-term camera trapping can address fundamental ecological knowledge gaps for cryptic and geographically restricted species. The resulting baseline information can support the detection of changes in occurrence, activity patterns, and temporal relationships, as well as long-term monitoring and conservation planning.

## Figures and Tables

**Table 1:** Observation counts for each of the studied species per season (seasons are pooled from three studied years).

Species/Category	Season				
	Spring	Summer	Autumn	Winter	Total
*Apodemus*	277	71	286	37	671
*Dinaromys*	144	133	246	59	582
*Eliomys*	7	33	21	0	61
Soricidae	31	20	60	48	159
Total	459	257	613	144	1473

**Table 2:** Comparisons of the activities of *D. bogdanovi* between seasons using the Wald test. Difference—difference between seasonal activity estimates; *p*—probabilities of accepting H0 (H0 is no difference between estimates). **Bold** values indicate significant difference.

Comparison	Difference	*p*
Spring–Summer	0.126	**0.015**
Spring–Autumn	0.032	0.608
Spring–Winter	0.142	**0.016**
Summer–Autumn	−0.094	0.117
Summer–Winter	0.016	0.775
Autumn–Winter	0.111	0.095

**Table 3:** Comparisons of the seasonal activities of *D. bogdanovi* and other selected species/groups using the Wald test. Difference—difference between seasonal activity estimates; *p*—probabilities of accepting H0 (H0 is no difference between estimates). **Bold** values indicate significant difference.

	Spring		Summer		Autumn		Winter	
	Difference	*P*	Difference	*P*	Difference	*p*	Difference	*p*
*Dinaromys–* *Eliomys*	0.290	**<0.001**	−0.006	0.920	0.301	**<0.001**		
*Dinaromys–Apodemus*	0.170	**<0.001**	0.031	0.552	0.067	0.292	0.132	**0.032**
*Dinaromys*– Soricidae	−0.080	0.388	−0.197	0.063	−0.096	0.290	−0.404	**<0.001**

## Data Availability

The data supporting the findings of this study are available from the corresponding author upon request. Upon acceptance, the dataset will be deposited in Figshare, and the repository link and digital object identifier will be added to the final published version.

## Appendix A

**Table A1:** Camera-trap sampling effort, expressed as camera-–days, by season and deployment period. One camera–day represents one camera operating for 24 h. Total sampling effort was 2504 camera–days.

Year	Camera–Days by Season				
	Spring	Summer	Autumn	Winter	Total
Deployment round 1					
2013	62	76	336	38	512
2014	168	198	350	81	797
2015	94	60	0	54	208
Total	324	334	686	173	1517
Deployment round 2					
2018	0	0	308	62	370
2019	270	196	31	120	617
Total	270	196	339	182	987
All rounds					
Total	594	530	1025	355	2504
